# LncRNA-TWIST1 Promoted Osteogenic Differentiation Both in PPDLSCs and in HPDLSCs by Inhibiting TWIST1 Expression

**DOI:** 10.1155/2019/8735952

**Published:** 2019-06-23

**Authors:** Yuerong Xu, Wen Qin, Donghui Guo, Jia Liu, Mingming Zhang, Zuolin Jin

**Affiliations:** ^1^State Key Laboratory of Military Stomatology and National Clinical Research Center for Oral Diseases and Shaanxi Clinical Research Center for Oral Diseases, Department of Orthodontics, School of Stomatology, The Fourth Military Medical University, Xi'an, Shaanxi 710032, China; ^2^Department of Cardiology, Tangdu Hospital, Fourth Military Medical University, Xi'an, China

## Abstract

HPDLSCs derived from periodontal ligament tissues contribute to tooth development and tissue regeneration. Exploring the effects of long noncoding RNAs (lncRNAs) in the process of osteogenic differentiation of periodontal ligament stem cells would provide novel therapeutic strategies for tissue regeneration. The expression levels of lncRNA, which significantly changed during osteogenic differentiation, were observed by real-time quantitative PCR (q-PCR). Then, we screened for osteogenic-related lncRNA, which was initially named lncRNA-TWIST1. Moreover, we detected the mRNA expression levels of TWIST1 and osteogenesis-related genes after upregulating and downregulating lncRNA-TWIST1 in PPDLSCs (periodontal mesenchymal stem cells from periodontitis patients) and HPDLSCs (periodontal mesenchymal stem cells from healthy microenvironment), respectively. The osteogenic degree was verified by detecting ALP activity and alizarin red staining. LncRNA-TWIST1 decreased the mRNA levels of TWIST1 and promoted osteogenic differentiation in PPDLSCs, which was confirmed by the increase in osteogenesis-related gene levels (Runx2, ALP, and OCN), the increase in ALP activity, and the formation of more osteogenic nodules. In contrast, downregulating lncRNA-TWIST1 decreased the expression of osteogenesis-related genes, ALP activity, and osteogenic nodules both in PPDLSCs and in HPDLSCs. LncRNA-TWIST1 promoted osteogenic differentiation both in PPDLSCs and in HPDLSCs by inhibiting the TWIST1 expression. LncRNA-TWIST1 may be a novel therapeutic strategy to regenerate dental tissues.

## 1. Introduction

HPDLSCs are derived from periodontal tissue. After being successfully isolated and cultured for the first time by Seo et al. in 2004 [1], hPDLSCs (human periodontal mesenchymal stem cells) have been considered the most important seed cells for healing periodontal defects. However, several factors have been shown to regulate the potency of hPDLSCs, including tissue origin [2], donor age [3], inflammatory condition [4], and growth factors [5]. Previous studies have confirmed that the inflammatory microenvironment decreases the osteogenic ability of hPDLSCs [4, 6, 7]. Therefore, exploring the factors that affect the osteogenic differentiation of PPDLSCs derived from an inflammation microenvironment and improving the osteogenic ability of PPDLSCs have become the focus and difficulty of current research studies.

In recent years, it has been found that lncRNAs (long noncoding RNAs), which are RNAs that exceed 200 bp in length and do not encode any protein, are involved in regulating the differentiation of mesenchymal stem cells [8, 9]. It has been reported that lncRNAs may be involved in osteogenesis [10].

In our previous study, we used the Arraystar human lncRNA chip (v3.0) to determine the different expression levels of lncRNAs and mRNAs between HPDLSCs and PPDLSCs. Ultimately, a total of 89 lncRNAs and 387 mRNAs were found (Change Fold> 2, P < 0.05) [11]. In this study, we selected out a lncRNA located on chromosome 7 from 19159555 to 19161539 with a length of 692 bp. We first named it lncRNA-TWIST1 because it is close to the TWIST1 gene. After upregulating or downregulating lncRNA-TWIST1, we detected changes in osteogenic potential at the cellular level and tested the expression levels of osteogenic genes. This research aims to provide a new target for restoring the osteogenic ability of PPDLSCs and improving therapies for periodontitis.

## 2. Methods

### 2.1. HPDLSC and PPDLSC Sample Selection and Primary Culture

HPDLSC tooth samples were taken from 10 healthy volunteers who were between 31 and 40 years old and who did not have periodontal diseases. There were no tooth loss and periodontal attachment loss in the healthy volunteers. PPDLSC tooth samples were taken from 10 volunteers with chronic periodontitis who were between the ages of 27 and 41. The periodontal pockets of tooth samples were deeper than 5 mm, and the alveolar bone resorption was more than 1/2 of the root length. Loss of periodontal attachment was ≥2mm and <5mm. There was no tooth loss in the volunteers with chronic periodontitis. None of these volunteers had acute periodontal infection, systemic disease, or a history of smoking, orthognathic surgery, radiotherapy, or chemotherapy. The tooth extraction was for an orthodontic treatment. All procedures were approved by the Ethical Committee of School of Stomatology, Fourth Military Medical University (IRB-REV-2015038). Meanwhile, the volunteers were informed of the experiment content and signed informed consent forms. We used collagenase type I and dispase to digest the tissues according to established protocols [12, 13]. The cells were cultured with *α*-MEM medium (GIBCO/Invitrogen, Carlsbad, CA, USA) supplemented with 10% fetal bovine serum (HyClone, Kerrville, TX, USA). To obtain single cell-derived colonies, single cells were seeded in each well of a 96-well plate and collected for passage until they reached 80% confluency. All experiments in this study were performed with 2nd- to 4th-generation cells.

### 2.2. Cells Immunophenotype

Based on established protocols, the PDLSCs were determined by stem cell surface markers and flow cytometry [12, 13]. HPLDSCs and PPDLSCs at the 4th passage were trypsinized into single cell suspensions and washed 3-4 times with PBS. The cell concentration was adjusted to 1×10^7^ cells/ml. Next, the cells were incubated with fluorescence-labeled antibodies (CD29, CD105, CD90, CD34, and CD45 (R&D Systems, USA)) at 4°C away from light for 1.5 h. After the cells were washed 3-4 times, the cells were detected with flow cytometry.

### 2.3. Real-Time PCR

Total RNA was isolated through the TRIZOL method. Additionally, the OD260/OD280 of mRNA was between 1.8 and 2.0. A reverse transcription kit (TAKARA, Osaka, Japan) was used for reverse transcription to obtain the first chain cDNAs. A standard SYBR Green PCR kit (TAKARA, Osaka, Japan) was used to amplify target fragments. The cDNA fragment of the *β*-actin gene was used as an internal control for the results analysis. Additionally, the mRNA levels of TWIST1, alkaline phosphatase (ALP), osteocalcin (OCN), and runt-related transcription factor 2 (Runx2) were detected. The sequences of the primers are shown in Table 1.

### 2.4. Lentivirus Infection

Construction of the lentivirus vector was performed by GeneChem (GeneChem, Shanghai, China). Lentiviruses: Ubi-MCS-SV40-EGFP-IRES-puromycin were used for lncRNA-TWIST1 overexpression; lentiviruses: hU6-MCS-CBh-gcGFP-IRES-puromycin were used for lncRNA-TWIST1 knockdown. The primers for amplifying lncRNA-TWIST1 had the following sequences: F:5′-ACCTAACAATTTTCTTTTCCAC-3′; R: 5′- GGCAGCTAAGACACCAACT-3′. The lncRNA-TWIST1 RNA interference targets had the following sequences: lncRNA-RNAi(58780-1), 5′-TTGCAAAGTCATCAGTCTCAA-3′; lncRNA-RNAi(58781-1), 5′-GAGCTAGATACTCCCTGCTTT-3′; lncRNA-RNAi(58782-1), 5′- AGGCTGGAGAGTTGGTGTCTT-3′; and NC, 5′- TTCTCCGAACGTGTCACGT-3′. HPDLSCs and PPDLSCs were transfected with lentiviruses (MOI: 50) to regulate the level of lncRNA-TWIST1. After 3 days, we detected the infection efficiency using an inverted fluorescence microscope (Olympus, Japan). The cells were collected for RNA extraction, and the infection efficiency and osteogenic gene expression were verified with real-time PCR. ALP staining and alkaline phosphatase activity tests were performed 7 days after using the osteoblast-inducing conditional media as the culture media. Additionally, real-time PCR was applied to detect the levels of osteogenic genes. Alizarin red staining and quantitative determination were performed 21 days after osteogenic induction.

### 2.5. Osteogenic Differentiation Assay

HPDLSCs and PPDLSCs were cultured in 6-well plates (1×10^5^ cells per well). The osteoblast-inducing conditional medium (a-MEM with 10% fetal bovine serum, *β*-glycerophosphate 10 mmol/L, ascorbic acid 50 g/ml, and dexamethasone 1 × 10 mol/L) was used. The osteoblast-inducing conditional medium was replaced every two days.

### 2.6. ALP Staining and ALP Activity Quantification

After inducing osteogenesis for 7 days, PPDLSCs were stained with ALP dye solution (Beyotime, China) at 37°C for 30 min. A week after osteogenic induction, the PPDLSCs were trypsinized. The working solution for the ALP activity assay was configured (Alkaline phosphatase assay kit, Nanjinganjing Jiancheng Bioengineering Institute, China). A suspension of the denatured cells and working solution was mixed in a 96-well plate and incubated for 30 min at 37°C. Next, the cells were treated with the chromogenic solution. A universal Microplate Spectrophotometer was used to detect the absorbance at a 520 nm wavelength.

### 2.7. Alizarin Red Staining

HPDLSCs and PPDLSCs were cultured in 6-well plates. When the cells reached 80% confluence, the induction medium for osteogenic differentiation was added to the cells to be cultured for another 3 weeks. Next, we used alizarin red staining solution (1 g alizarin red dissolved in 100 ml of distilled water) to stain the cells for 20 min. To quantify the alizarin red-stained nodules, the stain was solubilized and transferred to wells of a 96-well plate. Finally, the cells were quantified as previously described [14] with a Universal Microplate Spectrophotometer at a 520 nm wavelength.

### 2.8. Statistical Analysis

All experiments were performed in a blinded manner. The results were expressed as the means ± SEM. Statistical significance was determined by one-way ANOVA with Bonferroni correction for multiple comparisons or unpaired Student's t-tests. A value of P < 0.05 was considered statistically significant.

## 3. Results

### 3.1. Morphology of HPDLSCs and PPDLSCs

**Figure 1:**
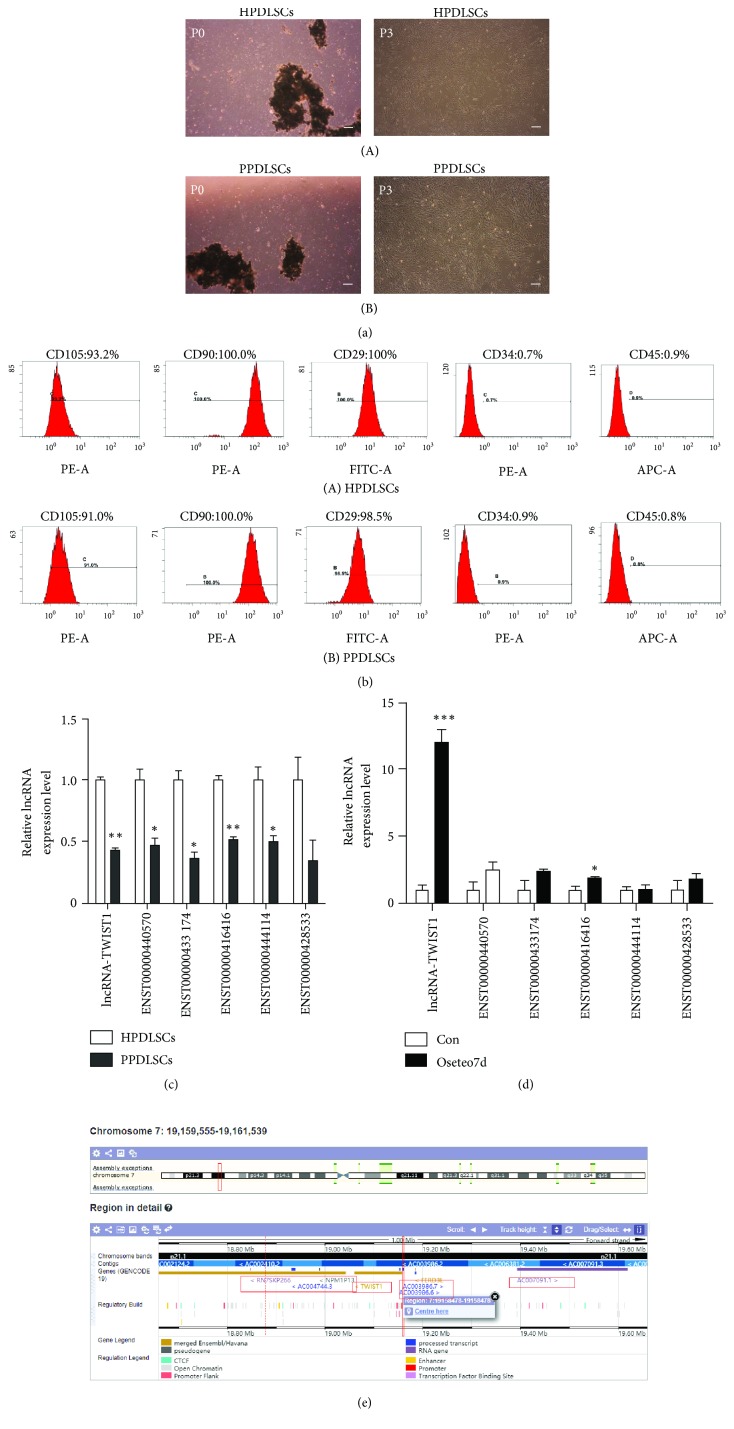
*LncRNA-TWIST1 was identified as the lncRNA that is related to osteogenic differentiation in PPDLSCs*. (a) Primary culture cells. P0: the primary generation. P3: the third generation. (b) The characterization of HPDLSCs (A) and PPDLSCs (B). The isolated cells expressed CD105, CD90, and CD29 (mesenchymal stem cell markers), but not CD45 (hematopoietic cell marker) and CD34 (leucocyte maker). (c) The difference in lncRNA expression levels between PPDLSCs and HPDLSCs. (d) The expression level of the lncRNAs in PPDLSCs after osteogenic differentiation for 7 days. (e) The location of the LncRNA-TWIST1 gene. Mean ± SEM (*∗*P< 0.05, *∗∗*P< 0.01).

Five to seven days after the primary culture, the cells began to crawl out of the tissue. The cells were spindle-shaped and could produce two or more pseudopodia. PPDLSCs were slenderer than HPDLSCs, which was more obvious in later passages, and they grew faster. HPDLSCs and PPDLSCs both could proliferate more after 10 stable passages (Figure 1).

### 3.2. Mesenchymal Stem Cell Surface Molecular Identification

We found that both HPDLSCs and PPDLSCs expressed CD105, CD90, and CD29 on the surface but did not express CD34 and CD45. Furthermore, the expression of CD105 and CD90 in HPDLSCs and PPDLSCs was almost 100% (Figure 1).

### 3.3. LncRNA-TWIST1 Was Identified as the lncRNA That Is Related to Osteogenic Differentiation in PPDLSCs

According to a previous study, we selected 6 lncRNAs that had high expression levels in HPDLSCs and were significantly downregulated in PPDLSC. To select osteogenic-related lncRNA, we planned to use real-time PCR to detect the changes in lncRNA expression levels in PPDLSCs after 7 days of induced osteogenesis. The results showed that lncRNA-TWIST1 was most obviously upregulated (10-fold) after osteogenic differentiation in PPDLSCs. Therefore, we made lncRNA-TWIST1 the target of our research (Figure 1).

### 3.4. The Expression Level of lncRNA-TWIST1 Increased in PPDLSCs during Osteogenic Differentiation

**Figure 2:**
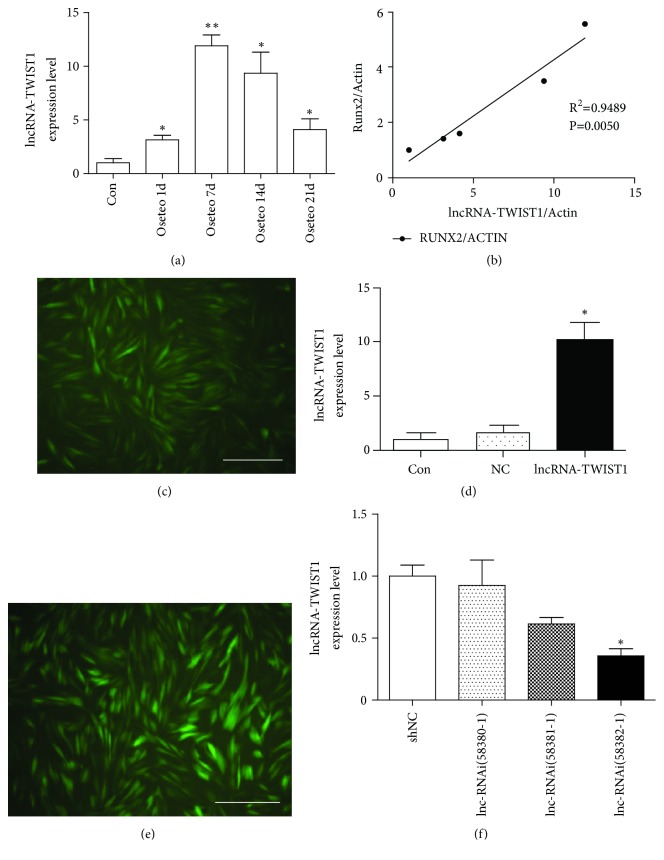
*The expression level of lncRNA-TWIST1 increased in PPDLSCs during osteogenic differentiation*. (a) The expression levels of lncRNA-TWIST1 at different osteogenic time points in PPDLSCs (*∗*P< 0.05, *∗∗*P< 0.01). (b) LncRNA-TWIST1 was highly correlated with the Runx2 during osteogenic differentiation (R2 = 0.9489). (c) PDLSCs transfected by an lncRNA-TWIST1-overexpressing lentivirus were observed under a fluorescence microscope (×400). (d) The efficiency of an lncRNA-TWIST1-overexpressing lentivirus was tested with real-time PCR (*∗*P<0.05). (e) PDLSCs transfected with an lncRNA-TWIST1-knockdown lentivirus were observed under a fluorescence microscope (×400). (f) The efficiency of the lncRNA-TWIST1-knockdown lentivirus was tested with real-time PCR (*∗*P<0.05). Mean ± SEM.

We observed the expression level of lncRNA-TWIST1 at 1 d, 7 d, 14 d, and 21 d after osteogenic induction of PPDLSCs to verify the role of lncRNA-TWIST1 during osteogenic differentiation. The expression level of lncRNA-TWIST1 increased on the first and the seventh day. Although the expression level of lncRNA-TWIST1 decreased on the fourteenth day and the twenty-first day, it was still higher than that in the con group. Meanwhile, we also observed the relationship between lncRNA-TWIST1 and mRNA-Runx2 expression at different time points after osteogenic induction of PPDLSCs. We found that, in the process of osteogenic differentiation, lncRNA-TWIST1 was highly correlated with Runx2 (the osteogenic marker) (R2 = 0.9489) (Figure 2).

### 3.5. LncRNA-TWIST1 Overexpressing Lentivirus Successfully Infected PPDLSCs

Three days after infection with an LncRNA-TWIST1-overexpressing lentivirus in PPDLSCs, we observed that green fluorescence-stained cells represented more than 80% of the well. Real-time PCR was used to check the infection efficiency. We found that lncRNA-TWIST1 expression in the PPDLSCs infected with an lncRNA-TWIST1 overexpressing lentivirus was increased up to 10.08 times more than PPDLSCs infected with an empty virus vector (Figure 2).

### 3.6. The lncRNA-TWIST1-Knockdown Lentivirus Was Successfully Transfected into PPDLSCs and HPDLSCs

To select high efficiency lncRNA-TWIST1-knockdown lentiviruses, we transfected three lentiviruses containing different lncRNA-RNAis, including lncRNA-RNAi(58380-1), lncRNA-RNAi(58381-1), and lncRNA-RNAi(58382-1). Real-time PCR showed that the lentivirus with lncRNA-RNAi(58380-1) had the highest inhibitory effect on lncRNA-TWIST1, and it was declared to be the lncRNA-TWIST1-knockdown lentivirus and used in subsequent experiments (Figure 2).

### 3.7. LncRNA-TWIST1 Upregulated the Osteogenic Differentiation of PPDLSCs In Vitro

**Figure 3:**
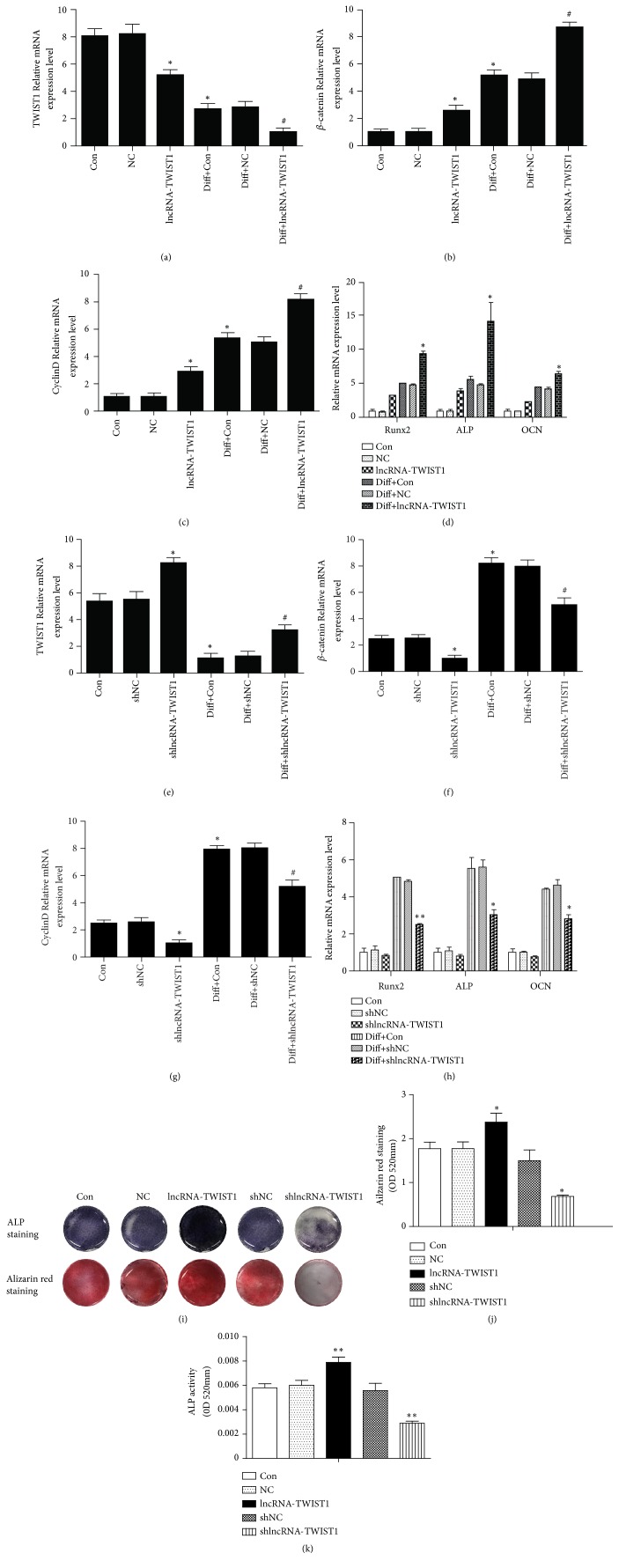
*LncRNA-TWIST1 inhibited the TWIST1 expression and upregulated the osteogenic differentiation of PPDLSCs*. (a–c) The mRNA levels of TWIST1, *β*-catenin, and cyclin D in PPDLSCs infected by the lncRNA-TWIST1-overexpressing lentivirus. (d) The mRNAs expression of osteogenic genes in PPDLSCs infected by the lncRNA-TWIST1-overexpressing lentivirus. (e–g) The mRNA expression levels of TWIST1, *β*-catenin, and cyclin D in PPDLSCs infected by the lncRNA-TWIST1-knockdown lentivirus. (h) The mRNAs expression of osteogenic genes in PPDLSCs infected by the lncRNA-TWIST1-knockdown lentivirus. (i) Alizarin red and ALP staining for osteogenic differentiations of PPDLSCs. (j) Quantitative measurement of Alizarin red staining. (k) ALP activity assay (*∗*P< 0.05, *∗∗*P< 0.01). Mean ± SEM.

After transfection with an lncRNA-TWIST1-overexpressing lentivirus, PPDLSCs underwent osteogenic induction for 7 days. We detected the expression levels of TWIST1 and osteogenic genes with real-time PCR, and we found that the levels of Runx2, ALP, and OCN were significantly upregulated and TWIST1 was downregulated. The function of Wnt/*β*-catenin signaling pathway in the regulation of the osteogenic differentiation is well known. We detected the Wnt/*β*-catenin pathway-related gene (*β*-catenin and cyclin D) by the real-time PCR. *β*-catenin and cyclin D mRNA expression levels were increased after lncRNA-TWIST1 treatment. ALP activity in the lncRNA-TWIST1-overexpressing group was increased, as demonstrated with the ALP staining. Twenty-one days after osteogenic induction, the lncRNA-TWIST1-overexpressing groups formed more calcium nodules than the control group in alizarin red staining. The results suggested that lncRNA-TWIST1 enhanced the osteogenic differentiation ability of PPDLSCs. The levels of Runx2, ALP, and OCN in PPDLSCs transfected with lncRNA-TWIST1-knockdown lentiviruses decreased significantly and TWIST1 was upregulated. *β*-catenin and cyclin D were decreased after downregulating the lncRNA-TWIST1. The ALP activity and the number of mineralized nodules also decreased. These results confirmed that lncRNA-TWIST1-knockdown inhibited osteogenic differentiation of PPDLSCs (Figure 3).

### 3.8. LncRNA-TWIST1-Knockdown Inhibited the Osteogenic Differentiation of HPDLSCs

**Figure 4:**
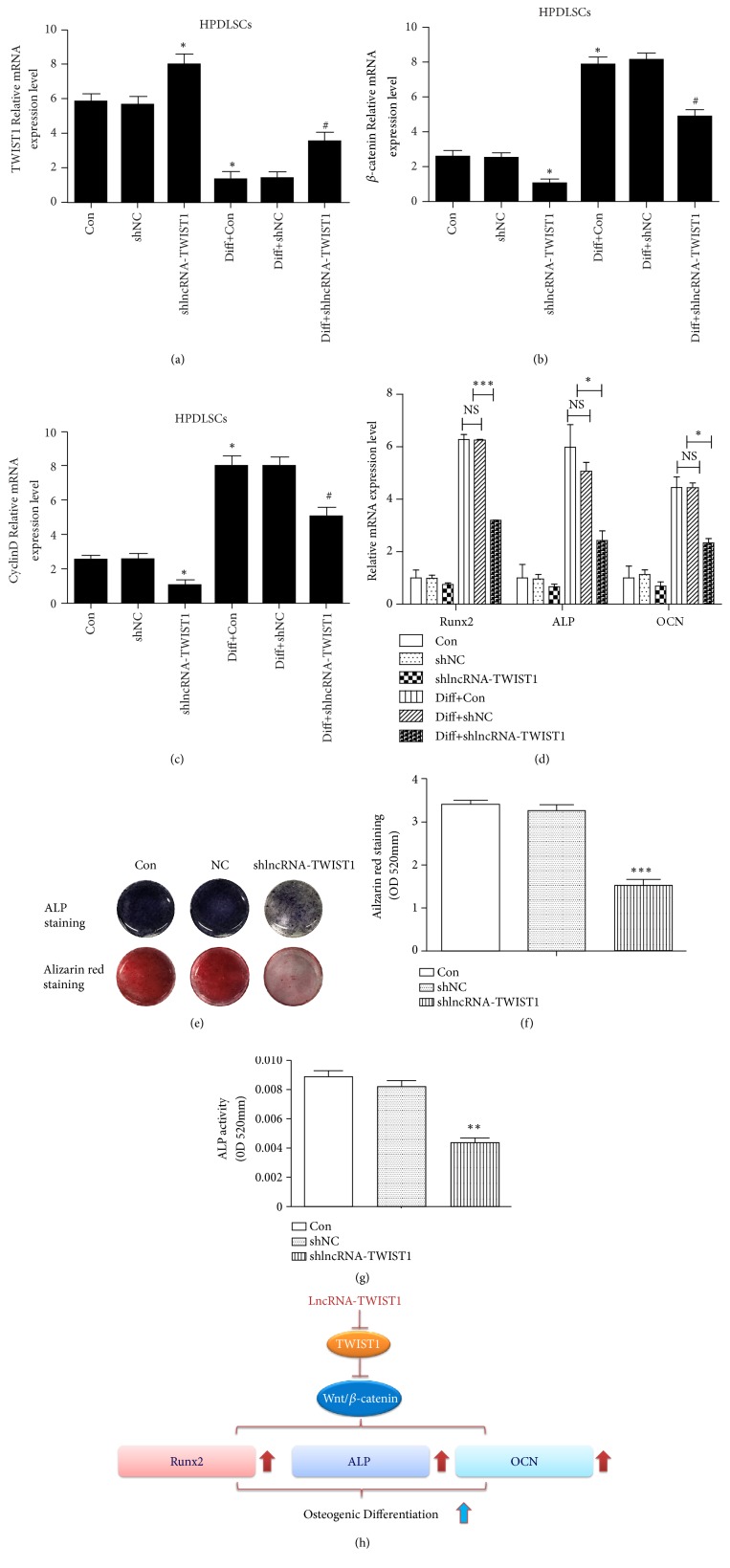
*LncRNA-TWIST1-knockdown increased the TWIST1 expression and inhibited the osteogenic differentiation of HPDLSCs*. (a–c) The mRNA levels of TWIST1, *β*-catenin, and cyclin D in HPDLSCs infected by lncRNA-TWIST1-knockdown lentivirus. (d) The mRNA expression of osteogenic genes in HPDLSCs infected by the lncRNA-TWIST1-knockdown lentivirus. (e) Alizarin red and ALP staining were used to detect osteogenic differentiation. (f) Quantitative measurement of Alizarin red staining. (g) ALP activity assay. (h) Schematic diagram depicting possible mechanisms involved in the effect of LncRNA-TWIST1 (*∗*P< 0.05, *∗∗*P< 0.01). Mean ± SEM.

To further investigate the role of lncRNA-TWIST1 during osteogenic differentiation, we observed the effects of lncRNA-TWIST1 knockdown in HPDLSCs. The levels of Runx2, ALP, and OCN decreased, and TWIST1 expression increased when we inhibited the lncRNA-TWIST1 expression in HPDLSCs. *β*-catenin and cyclin D were decreased after downregulating the lncRNA-TWIST1. In addition, the results of ALP staining, ALP activity assay, and alizarin red staining also support the conclusion that downregulating the lncRNA-TWIST1 expression in HPDLSCs will reduce the osteogenic differentiation of HPDLSCs (Figure 4).

## 4. Discussion

Periodontitis is a chronic infection of periodontal tissue caused by bacteria, and it leads to alveolar bone absorption and is the main cause of tooth loss in adults [15]. Dentists have been able to successfully heal inflammation in their clinics. However, determining how to repair damaged periodontal tissue is still the focus of many current research efforts. Three elements of periodontal regeneration are stem cells, growth factors, and extracellular matrix scaffolds [16–18].

PDLSCs contribute to the formation of adjacent bone tissues [19, 20]. PDLSCs are widely recognized as the most promising seed cells for periodontal regeneration [16–18]. However, Liu Na et al. found that PPDLSCs formed fewer osteogenic nodules than HPDLSCs and had low Runx2, ALP, and OCN expression levels [21]. They have concluded that the loss of pluripotency of PPDLSCs is the most important reason for periodontal destruction. Furthermore, the impaired multidifferentiation function of PPDLSCs may be irreversible. In our research, we also observed that PPDLSCs have a lower osteogenic differentiation ability than HPDLSCs.

As the focus of current research, lnc-RNAs have a very wide range of biological functions [22], including chromosomal inactivation [23] and differentiation [8], pluripotent reprogramming [24], and apoptosis [25]. Lnc-RNAs also regulate the pathological processes of many diseases, including neurological diseases, cardiovascular diseases, inflammation, and cancer [26]. The osteogenic differentiation of MSCs could be regulated by lnc-RNAs. Huang et al. found that H19 could promote the osteogenic differentiation of MSC [10]. It has also been shown that lncRNA-ANCR can inhibit osteogenic differentiation of HPDLSCs [27]. Additionally, lncRNA-ANCR can directly bind to EZH protein to inhibit Runx2 expression, which is a key transcription factor in osteogenic differentiation that results in decreased osteogenic differentiation of MSCs [28].

To clarify the function of lncRNA in osteogenic differentiation of PPDLSCs, we used the Arraystar human LncRNA chip (v3.0) to exam the different expression of lncRNA between PPDLSCs and HPDLSCs. Additionally, 89 lncRNAs and 387 mRNAs have different expression levels (Change Fold> 2, P <0.05) [11]. The biological function of lncRNA-TWIST1 has never been elucidated in other studies. We demonstrated that lncRNA-TWIST1 regulated osteogenic differentiation of PPDLSCs. Additionally, lncRNA-TWIST1 was significantly downregulated in PPDLSCs compared to HPDLSCs and increased the process of osteogenesis that is highly correlated with Runx2, which indicates that the downregulation of lncRNA-TWIST1 may be one of the reasons for the impaired osteogenic ability of PPDLSCs.

TWIST1 is identified as a highly conserved protein [29]. It has been suggested that Twist inhibits Runx2 expression in the process of skeletogenesis [30, 31]. A previous study indicated that removing TWIST1 enhanced the osteogenic ability of hASCs. LncRNA-TWIST1 is close to TWIST1. LncRNA-TWIST1 may regulate TWIST1 expression. Wnt/*β*-catenin signaling pathway is involved in the osteogenic differentiation [32]. In our study, we found that lncRNA-TWIST1 overexpression inhibited TWIST1 expression, which suggests that lncRNA-TWIST1 regulates TWIST1 expression in PPDLSCs. LncRNA-TWIST1 activated the Wnt/*β*-catenin signaling pathway to promote the osteogenic differentiation of PPDLSCs.

Runx2 is a key component of collagen, and thus the mRNA expression of Runx2 represents osteogenic differentiation to a certain degree. We found that lncRNA-TWIST1 expression during osteogenic differentiation is highly correlated with Runx2, which suggests lncRNA-TWIST1 may be involved in the regulation of osteogenic differentiation of PPDLSCs.

Next, we constructed lncRNA-TWIST1 overexpression and a knockdown lentivirus to upregulate or downregulate lncRNA-TWIST1 as well as conducting a series of experiments. After inducing osteogenic differentiation, the expressions of Runx2, ALP, and OCN in PPDLSCs, which are the osteogenic genes, were significantly increased, which indicated there was successful osteogenic induction. The level of osteogenic gene mRNAs in the lncRNA-TWIST1 overexpression group was obviously increased compared to the control group, whereas the lncRNA-TWIST1 knockdown group did not change. The results of alkaline phosphatase activity assay and staining as well as alizarin red staining showed that the osteogenic ability of the lncRNA-TWIST1 upregulating group increased, whereas the lncRNA-TWIST1 knockdown group had a decreased ability.

HPDLSCs are a kind of mesenchymal stem cell and could differentiate into multiple cell types under different conditions [33, 34]. Since HPDLSCs are specially derived from periodontal ligaments and are involved in the formation of adjacent bone tissues [19, 20], HPDLSCs are widely recognized as the most promising seed cells of periodontal regeneration. However, HPDLSCs in the chronic periodontitis tissue have the worst osteogenic ability, which is the most important reason for periodontal destruction. For the first time in 2011, Liu Na et al. found that PPDLSCs formed fewer osteogenic nodules than HPDLSCs and had low Runx2, ALP, and OCN mRNA expression [21]. To further clarify the function of lncRNA-TWIST1 in the osteogenic differentiation of HPDLSCs, we also observed the effects of the lncRNA-TWIST1-knockdown lentivirus on HPDLSCs, and we found that lncRNA-TWIST1-knockdown significantly inhibited the osteogenesis of HPDLSCs. These results confirmed that lncRNA-TWIST1 promoted the osteogenic differentiation of PPDLSCs. LncRNA-TWIST1 may be a new target for repairing damaged periodontal tissue. However, these conclusions are based on in vitro experiments. Whether lncRNA-TWIST1 promotes osteogenic differentiation in vivo requires further investigation and verification.

In conclusion, LncRNA-TWIST1 promoted osteogenic differentiation both in PPDLSCs and in HPDLSCs by inhibiting the TWIST1 expression. LncRNA-TWIST1 may be a novel therapeutic strategy for regenerating dental tissues.

## Figures and Tables

**Table 1:** Primer Sequences.

Gene	Primer sequences (5' to 3')
ACTB	F:5- TCAAGATCATTGCTCCTCCTGAG -3'
	R:5- ACATCTGCTGGAAGGTGGACA -3'
Runx2	F: 5'- CCCGTGGCCTTCAAGGT -3'
	R: 5'- CGTTACCCGCCATGACAGTA -3'
ALP	F: 5'- GGACCA TTCCCACGTCTTCAC -3'
	R: 5'- CCTTGTAGCCAGGCCCATTG -3'
OCN	F: 5'- CTCACTCTGCTGGCCCTGAC -3'
	R: 5'- CCTTACTGCCCTCCTGCTTG -3'
ENST0000041TWIST1	F: 5'- CCTAACCAGAACCATCCTGCC -3'
	R: 5'- CAAAAGTCGTCTCATCCTCCAC -3'
ENST00000440570	F: 5'- GCACACAGACAAGAACTAAAGTGG -3'
	R: 5'- TGGACAGTTGCCCATATTAACG -3'
ENST00000433174	F: 5'- ATGAGTTATGAGGTGAAGGAGGG -3'
	R: 5'- CTGCTTGTTGCCTTAGTTTCTTC -3'
ENST00000416416	F: 5'- ATCACTATTGCCCATGTGGC -3'
	R: 5'- TGTTGGCTACCTCATACTTGCTG -3'
ENST00000444114	F: 5'- CTGGAATTACTGGAATCACACTGTC -3'
	R: 5'- CTCAGACCATCCATCGCTCC -3'
ENST00000428533	F: 5'- CCATCAGGAAGCAGAGAACAAG -3'
	R: 5'- AGGGTCTCTGAACCGCACTT -3'
TWIST1	F: 5'- AGCTACGCCTTCTCGGTCT-3'
	R: 5'- CCTTCTCTGGAAACAATGACATC-3'
*β*-catenin	F: 5'- CTTCACCTGACAGATCCAAGTC-3'
	R: 5'- CCTTCCATCCCTTCCTGTTTAG-3'
Cyclin D	F: 5'- GTTCGTGGCCTCTAAGATGAAG-3'
	R: 5'- GATGGAGTTGTCGGTGTAGATG-3'

## Data Availability

The research article data used to support the findings of this study are included within the article.
